# Forecasting Malaria Cases Using Climatic Factors in Delhi, India: A Time Series Analysis

**DOI:** 10.1155/2014/482851

**Published:** 2014-07-23

**Authors:** Varun Kumar, Abha Mangal, Sanjeet Panesar, Geeta Yadav, Richa Talwar, Deepak Raut, Saudan Singh

**Affiliations:** Department of Community Medicine, Vardhman Mahavir Medical College and Safdarjung Hospital, New Delhi 110029, India

## Abstract

*Background*. Malaria still remains a public health problem in developing countries and changing environmental and climatic factors pose the biggest challenge in fighting against the scourge of malaria. Therefore, the study was designed to forecast malaria cases using climatic factors as predictors in Delhi, India. *Methods*. The total number of monthly cases of malaria slide positives occurring from January 2006 to December 2013 was taken from the register maintained at the malaria clinic at Rural Health Training Centre (RHTC), Najafgarh, Delhi. Climatic data of monthly mean rainfall, relative humidity, and mean maximum temperature were taken from Regional Meteorological Centre, Delhi. Expert modeler of SPSS ver. 21 was used for analyzing the time series data. *Results*. Autoregressive integrated moving average, ARIMA (0,1,1) (0,1,0)^12^, was the best fit model and it could explain 72.5% variability in the time series data. Rainfall (*P* value = 0.004) and relative humidity (*P* value = 0.001) were found to be significant predictors for malaria transmission in the study area. Seasonal adjusted factor (SAF) for malaria cases shows peak during the months of August and September. *Conclusion*. ARIMA models of time series analysis is a simple and reliable tool for producing reliable forecasts for malaria in Delhi, India.

## 1. Introduction

Every year, more than 1 billion people are infected and more than 1 million die from vector-borne diseases. World Health Organization (WHO) has highlighted the serious and increasing threat of vector-borne diseases with the theme “Preventing vector-borne diseases” and also with the slogan “Small bite, big threat” for the year 2014. Among vector-borne diseases, malaria poses the biggest threat with about 40% of the world's population at risk of infection. In 2013, 97 countries had ongoing transmission of malaria [1]. Malaria decreases economic growth by more than one percentage point per year in endemic countries. Malaria transmission season generally coincides with the harvesting season and brief periods of illness exact a high cost on the world's poorest regions [2].

In spite of various recent advancements in diagnostic and treatment modalities, malaria still remains a public health problem in developing countries and changing environmental and climatic conditions are considered as the biggest challenge in fighting against the scourge of malaria [3]. Malaria is an entirely preventable and treatable illness caused by parasites of* Plasmodium* species and transmitted exclusively by the bites of* Anopheles* mosquito. Although preventable and treatable, malaria causes significant morbidity and mortality, particularly in resource poor regions. It was estimated that 3.4 billion people are at risk of malaria in 2013. There were an estimated 207 million cases of malaria and an estimated 627 000 deaths in 2012 [4].

In South East Asia, which is the second most affected region by malaria in the world, India has the maximum number of cases with an estimated 24 million cases per year. Unlike Africa, where most of the deaths are reported in infants and children, in India, it is seen that malarial mortality is maximum in the economically productive age groups of 15 to 44 years [5]. A malaria-stricken family spends an average of over one quarter of its income on malaria treatment, as well as paying prevention costs and suffering loss of income [6]. Due to the severe health impact of malaria, there is a growing need for methods that will allow forecasting and early warning with timely case detection in areas of unstable transmission, so that more control measures can be implemented effectively [7].

Studies of malaria epidemics have shown their association with climatic conditions like rainfall patterns, temperature, and humidity. In many places, transmission is seasonal, with the peak during and just after the rainy season [8]. Malaria infections are often more common during rainy seasons because of increase in number of breeding sites. Optimal conditions for malaria transmission occur when the temperature is between 20°C and 30°C and the mean relative humidity is at least 60%. Water temperatures regulate the duration of aquatic breeding cycle of the mosquito vector and high relative humidity increases mosquito longevity [9].

Although there has been marked reduction in the number of malaria cases in India under National Vector Borne Disease Control Programme (NVBDCP), malaria still is the leading cause of infectious diseases with the development of drug resistant* Plasmodium* species and insecticide resistant mosquitoes. Forecasting of malaria cases allows for allocation of appropriate resources to target prevention and treatment of malaria and also to plan for eventual elimination. Malaria incidence in a particular month can be predicted by rainfall, temperature, and relative humidity [8]. Therefore, the present study was designed with objectives of developing a temporal model for forecasting malaria cases using climatic factors such as rainfall, relative humidity, and mean maximum temperature as predictors in Delhi, India.

## 2. Materials and Methods

### 2.1. Study Area

The study was conducted at the Rural Health Training Centre (RHTC), Najafgarh, which is a field practice area of the Department of Community Medicine, Vardhman Mahavir Medical College and Safdarjung Hospital. It is located in South-West District of Delhi. According to 2011 census the total population of Najafgarh was 906 452 [10]. The study area is situated at an altitude of 216 metres above sea level and has a monsoon influenced humid subtropical climate with high variation between summer and winter temperature and precipitation. Monsoon in Delhi starts in late June and extends till mid-September [11].

### 2.2. Data Collection

The total number of monthly cases of malaria slide positives from January 2006 to December 2013 was taken from the register maintained at the malaria clinic located at RHTC Najafgarh. This is the only malaria clinic located in the catchment area of RHTC Najafgarh, New Delhi, and it captures almost all malaria cases in that area. The data for mean rainfall, relative humidity (at 08:30 IST), and mean maximum temperature for the corresponding months were taken from Regional Meteorological Centre, Delhi. Since the data was collected from official registers and no personal information of any kind was obtained, ethical clearance was not sought.

### 2.3. Statistical Analysis

Expert modeler of SPSS ver. 21 software was used to fit the best suitable model for the time series data. The stationarity of the data was checked by autocorrelation function (ACF) and partial autocorrelation function (PACF). Seasonal adjusted factor (SAF) was used to determine the peak of seasonal variation. The Ljung-Box (modified Box-Pierce) test was used to determine if the model was correctly specified. To address the confounding factors, forecasting of the incidence of monthly malaria cases was done including the climatic predictors using the best fit model.

## 3. Results

**Figure 1:**
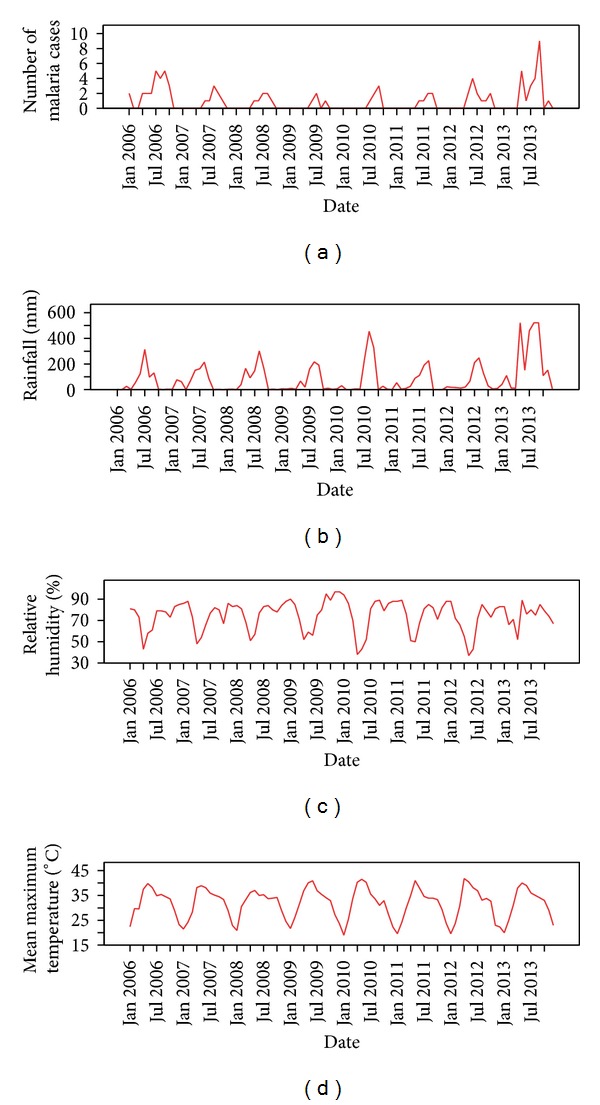
Monthly malaria cases, rainfall, relative humidity, and mean maximum temperature from January 2006 to December 2013 in the study area.

The total number of monthly laboratory confirmed cases of malaria showed a declining trend from the year 2006 to 2011 (Table 1). But it increased during the years 2012 and 2013 due to increase in the amount of rainfall received as shown in Figure 1.

Exploration of the monthly malaria infections, rainfall pattern, mean maximum temperatures, and relative humidity from 2006 to 2013 shows no clear trend and suggests a seasonal dependency in the series. All series exhibited number of peaks besides small scale fluctuations. The significant peaks in the series created by monthly malaria infections appeared to be separated by more than a few months showing a cyclical seasonal pattern as the peak of malaria infections follows a similar pattern with an interval of few months between the peaks (Figure 1).

The autocorrelation function (ACF) for the series by monthly malaria infections showed a significant peak at a lag of 12 months (autocorrelation = 0.675; Box-Ljung statistics (*P* = 0.000)) suggesting the presence of a seasonal component in the data. Partial autocorrelation function (PACF) also showed a significant peak at a lag of 12 which confirmed the presence of seasonal component in the time series data.

Observations without seasonal variation have a seasonal component of 0.  Table 2 shows that from the month of June to September, the seasonal adjustment factor (SAF) of malaria was more than 1; that is, in these months the malarial infections were more above the typical months. This period also coincides with the monsoon season in Delhi. Among these months, August had the highest SAF of 3.14 followed by September which had SAF of 3.1. This shows that the peak of malaria transmission occurs during these months where the risk is 3.14 times and 3.1 times higher than a typical month.

**Figure 2:**
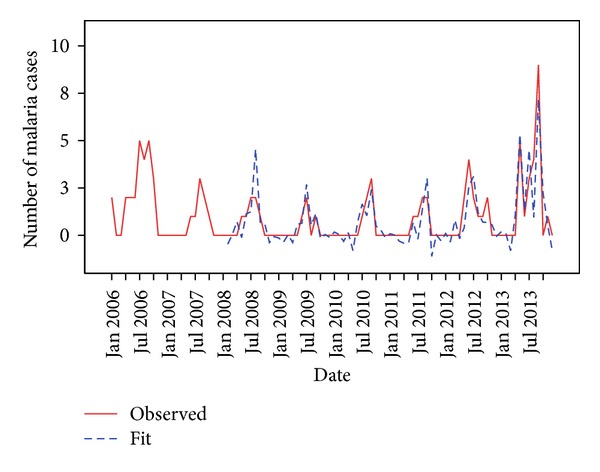
Actual (observed) and predicted (fit) values of malaria cases.

Expert modeler of SPSS ver. 21 was used to find the best fit model for forecasting malaria cases using mean rainfall, relative humidity, and mean maximum temperature, all lagged at one month as covariates. It suggested autoregressive integrated moving average model, ARIMA (0,1,1) (0,1,0)^12^ as the best fit statistical model for this time series data. It was found that only two climatic factors, mean rainfall (*P* value = 0.004), and relative humidity (*P* value = 0.001) both lagged at one month were significant predictors of malaria infections in the study area. Mean maximum temperature does not significantly predict malaria infections. The actual observed values and the predicted values matched reasonably well (Figure 2).

The Ljung-Box (modified Box-Pierce) statistics indicated that the model was correctly specified (Table 2). ARIMA (0,1,1) (0,1,0)^12^ model detected two outliers in the data. Although the time series modeler offers a number of different goodness-of-fit statistics, here stationary *R*-squared value was used since it provides an estimate of the proportion of the total variation in the series that is explained by the model. It is preferable to ordinary *R*-squared when there is a trend or seasonal pattern as is the case here. Larger values of stationary *R*-squared (up to a maximum value of 1) indicate better fit. A value of 0.725 meant that the model could explain 72.5% of the observed variation in the series (Table 3).

The forecasting model proposed, ARIMA, provides a comprehensive set of tools for univariate time series model identification, parameter estimation, and forecasting, and it offers great flexibility in analysis, which has contributed to its popularity in several areas of research and practice. A seasonal ARIMA model is represented by ARIMA (*p*, *d*, *q*)(*P*, *D*, *Q*)^*s*^ where *p* and *P*- are the autoregressive and seasonal autoregressive, respectively, *d* and *D*- are the nonseasonal differences and seasonal differencing, respectively, *q* and *Q*- are the moving average parameters and seasonal moving average parameters, respectively, and *s* represents the length of the seasonal period.

**Figure 3:**
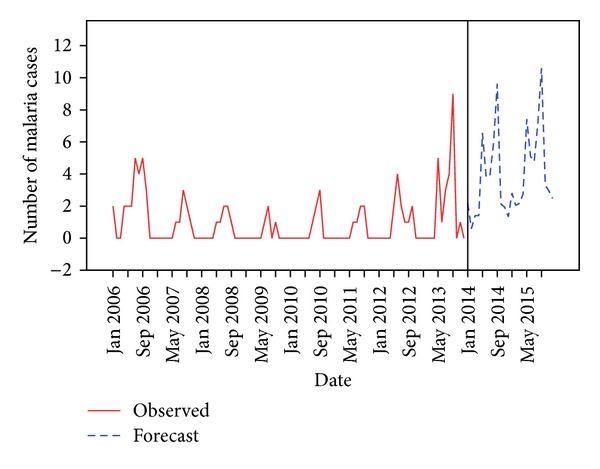
Observed and forecasted values for malaria cases.

ARIMA (0,1,1) (0,1,0)^12^ model was used to forecast the monthly malaria cases for the future from January 2014 to December 2015. This model also included the significant predictors for malaria cases, rainfall, and relative humidity which were lagged at one month. The forecasted model predicted that there will be a total of 41 cases of malaria in 2014 and 53 cases in 2015. The forecasted cases also show a seasonal pattern with significant peaks during rainy season (Figure 3).

## 4. Discussion

ARIMA models are useful in modeling the temporal dependence structure of a time series as they explicitly assume temporal dependence between observations and a useful tool in epidemiological surveillance. They are particularly useful for diseases which show a seasonal pattern [12]. ARIMA (0,1,1) (0,1,0)^12^ model developed in this study attempts to provide a simple tool to predict the expected number of malaria cases per month in the future based on observed malaria cases over the years and combination of climatic factors; in this study, rainfall and relative humidity were found to be significant predictors. These climatic covariates were lagged at one month because* Anopheles* vector takes two weeks to complete their life cycle and additional two more weeks for the generation of parasites in the new host. Wangdi et al. have also found ARIMA (2,1,1) (0,1,1)^12^ to be the best possible model for predicting malaria cases in Bhutan [13]. ARIMA model was also used for forecasting malaria cases in Sri Lanka [14] and Ethiopia [15].

Malaria transmission was found to occur between the months of July and September with a peak in the month of August. Rainy season in Delhi starts in late June and lasts till mid-September. This shows that the malaria incidence in Delhi has a strong correlation with rainfall in the preceding month. Loevinsohn ME has also found similar results in Rwanda [16]. Rainfall increases the number of vector breeding places which favors malaria transmission. It is also interesting to find that the maximum number of cases occur during the months of August and September and decreases thereafter. Continuous and heavy rainfall washes off the vector breeding places. Though rainy season in Delhi begins in late June, it receives heavy rainfall during August and September. This explains the decrease in the number of malaria cases after September.

Malaria transmission also depends on relative humidity since mosquitoes have a limited range of tolerable relative humidity. The high surface area to volume ratio of mosquitoes makes them especially sensitive to desiccation at low humidity levels [17]. Transmission of malaria occurs when the relative humidity is at least 60%. In the present study, relative humidity was found to be a strong predictor for forecasting malaria cases. Relative humidity in Delhi remains above 60% in the peak transmission season except during the summer months of April and May during which the transmission of malaria was also found to be low.

Apart from rainfall and relative humidity, temperature also plays a significant role in malaria transmission. Temperature rise is expected to increase transmission and prevalence of malaria by shortening the incubation period of the parasite in the mosquitoes. Sporogonic cycles take about 9 to 10 days at temperatures of 28°C but higher than 30°C and below 16°C have negative impact on parasite development [18]. But in the present study, mean maximum temperature was not found to be a significant predictor of malaria incidence unlike studies done in Bhutan [13] and Rwanda [16]. The mean maximum temperature during summers in Delhi is above 40°C which is unsuitable for vector breeding unlike Bhutan where the mean maximum temperature is around 35°C [13].

Apart from climatic factors, various other factors like the type of vector, the type of parasite, population movement and migration, urbanization, the level of immunity to malaria in the human hosts, insecticide resistance in mosquitoes, and drug resistance in parasites all play a significant role in affecting the severity and incidence of malaria. The statistical model developed in this study assumes that these factors remain constant over a period of time, taking only climatic predictors to forecast malaria incidence.

Based on the results of the present study, we believe that malaria transmission in Delhi depends on rainfall and relative humidity. The results also indicate that the malaria cases will continue to occur in the near future if appropriate actions are not initiated on time. The potential implication of this study is that by developing forecasting models for predicting the expected number of malaria cases in advance, timely prevention and control measures can be effectively planned like eliminating vector breeding places, spraying insecticides, and creating public awareness months before the peak season. The study also provides a model to foresee and allocate appropriate resources during peak transmission seasons. The time series model used in this study can also be applied to other diseases like Dengue. This result can also be used to inform travelers about malaria risk and screening and to take necessary precautionary measures.

The study also has some limitations. The study assumes that the reporting and registering of monthly malaria cases remain the same throughout the study period. Since the study was done by collecting data from a single centre, it is difficult to generalize the results in the actual population. The study can be done on a wider area and further research can be done to evaluate the effectiveness of integrating the forecasting model into the existing disease control program in terms of its impact in reducing the disease occurrence.

## 5. Conclusion

A seasonal pattern was observed in malaria incidence in Delhi with variable amplitudes of fluctuation with peak during the months of August and September. ARIMA (0,1,1) (0,1,0)^12^ model was found to be the best fit statistical model for predicting malaria cases in Delhi. Rainfall and relative humidity were found to be strong predictors of malaria infection in the study area. This information will also be useful for administrators in effectively implementing preventive and control measures for malaria.

## Figures and Tables

**Table 1:** Monthly malaria cases at RHTC Najafgarh, Delhi, during the study period (*n* = 92).

Month	2006	2007	2008	2009	2010	2011	2012	2013
January	2	0	0	0	0	0	0	0
February	0	0	0	0	0	0	0	0
March	0	0	0	0	0	0	0	0
April	2	0	0	0	0	0	0	0
May	2	0	1	0	0	0	2	5
June	2	1	1	1	0	1	4	1
July	5	1	2	2	1	1	2	3
August	4	3	2	0	2	2	1	4
September	5	2	1	1	3	2	1	9
October	3	1	0	1	0	0	2	0
November	0	0	0	0	0	0	0	1
December	0	0	0	0	0	0	0	0

Total	25	8	7	5	6	6	12	23

**Table 2:** Seasonal adjustment factor (SAF) for malaria cases.

Month	Observed cases	SAF
January	2	0
February	0	0
March	0	0
April	2	0
May	10	0.80
June	11	1.89
July	17	2.41
August	18	3.14
September	24	3.10
October	7	0.66
November	1	0
December	0	0

**Table 3:** Model statistics for malaria data.

Model parameter	Stationary *R* ^2^	Ljung-Box statistic			Model type
		statistics	df	*P* value	
Monthly malaria infections	0.725	22.899	17	0.653	ARIMA (0, 1, 1) (0, 1, 0)^12^
